# Development of Radio-Frequency Sensor Wake-Up with Unmanned Aerial Vehicles as an Aerial Gateway

**DOI:** 10.3390/s19051047

**Published:** 2019-03-01

**Authors:** Jianfei Chen, Zhaohua Dai, ZhiQiang Chen

**Affiliations:** 1 Department of Computer Science Electrical Engineering, University of Missouri-Kansas City, Kansas City, MO 64110, USA; jc6c2@mail.umkc.edu; 2 School of Civil Engineering, Nanjing Forestry University, Nanjing 210037, China; daizhaohua@njfu.edu.cn; 3 School of Computing and Engineering, University of Missouri-Kansas City, Kansas City, MO 64110, USA

**Keywords:** unmanned aerial vehicle, aerial–ground network, energy efficiency, sensor activation, radio frequency

## Abstract

The advent of autonomous navigation, positioning, and general robotics technologies has enabled the improvement of small to miniature-sized unmanned aerial vehicles (UAVs, or ‘drones’) and their wide uses in engineering practice. Recent research endeavors further envision a systematic integration of aerial drones and traditional contact-based or ground-based sensors, leading to an aerial–ground wireless sensor network (AG-WSN), in which the UAV serves as both a gateway besides and a remote sensing platform. This paper serves two goals. First, we will review the recent development in architecture, design, and algorithms related to UAVs as a gateway and particularly illustrate its nature in realizing an opportunistic sensing network. Second, recognizing the opportunistic sensing need, we further aim to focus on achieving energy efficiency through developing an active radio frequency (RF)-based wake-up mechanism for aerial–ground data transmission. To prove the effectiveness of energy efficiency, several sensor wake-up solutions are physically implemented and evaluated. The results show that the RF-based wake-up mechanism can potentially save more than 98.4% of the energy that the traditional duty-cycle method would otherwise consume, and 96.8% if an infrared-receiver method is used.

## 1. Introduction

With advances in autonomous navigation, positioning, and in general robotics technologies, small to miniature-sized unmanned aerial vehicles (UAVs, or colloquially called drones) are witnessing their ever-increasing use in engineering practice. Small UAVs are low-cost, agile, and flexible in imaging payloads as remote sensing platforms when compared with traditional space- or airborne platforms (e.g., satellites or aircrafts) [1]. Today’s UAVs have incorporated the latest GPS technology. Many small UAVs, especially the multi-motor ones, can fly following predetermined GPS waypoints. Some advanced drones have been equipped with lost-cost radar or vision sensors, acquiring a minimum level of flying beyond (visual) line-of-sight (BVLOS or BLOS) due to their sense-and-avoid capabilities [2,3,4]. This potentially would further render small UAVs an attractive remote sensing platform for numerous different applications.

On the other hand, wireless sensing network (WSN) technology has matured in recent years, with applications found in many scientific and engineering projects. Many WSN applications focus on ad-hoc tasks, wherein the local sensors are deployed with a goal of completing the task in a short time. Therefore, the energy consumption is not excessive. However, for deploying WSNs over a geospatially large or spatially complex space with an expected long-term monitoring mission, both WSN implementation and energy efficiency become the primary challenges. One possible application of sensing is in farming land, wherein precision agriculture (PA) practice demands data sensing at different granular (spatial and temporal) scales [5]. Another application scenario is performing structural health monitoring (SHM) for civil structures and lifeline infrastructure systems that are often massive and spatially complex (e.g., urban buildings, long-span bridges, and power transmission lines/towers, etc.). For structures that are critical to society, sensors, and especially WSNs, can be installed in these structures in their lifetimes to achieve ‘smart structures’. Continuous health monitoring through these WSNs provides stakeholders a basis for ensuring public safety and for decision-making when dealing with unexpected natural or technological disruptions [6].

**Figure 1:**
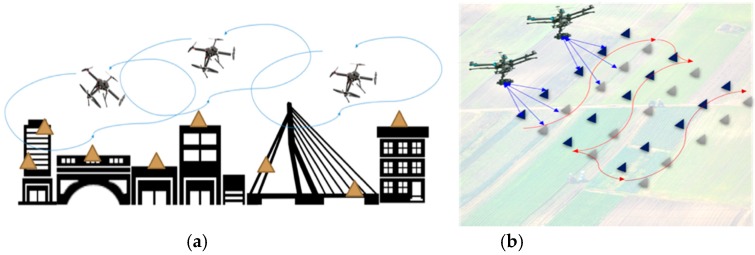
Conceptual illustrations of the proposed aerial–ground wireless sensing network (AG-WSN) for field monitoring: (**a**) complex 3D field in an urban setting and (**b**) largely 2D terrain field in an agriculture setting.

Taking the two areas of PA and SHM as the application settings (Figure 1) it is asserted that, in both situations, the necessity of combining UAV-based remote sensing and WSNs is straightforward. In PA, the traditional practice relies on sensing data such as space- or airborne imagery for decision-making and management in farming [7,8,9]. However, the high cost and the long revisit period of satellite or aerial imagery may prevent applying precision agriculture solutions at any location and time around the world. Images taken by low altitude UAVs give an alternative solution in the emerging precision agriculture practice [10,11,12]. In addition, since microelectromechanical systems (MEMS) technology and, particularly, the emerging Internet of Things (IoT) sensing technology have been rapidly improved in recent decades, many researchers have proposed and implemented different ground-based wireless sensor solutions for facilitating precision agriculture [13,14,15,16,17]. To achieve data fusions and more intelligent and tactical operations in these sensing modalities, integration of UAVs and ground-based WSNs becomes a rational choice. In the arena of SHM, the traditional approach is to deploy wired or wireless sensors to obtain the real-time responses of structures to environmental or hazard-induced vibrations [18,19,20,21]. In reality, however, for structures with slight to moderate damage, such as local cracking and corrosion, visual or remote sensing-based inspection is the most efficient approach to date. In recent years, as UAV technology has penetrated into many industrial sectors, small UAVs have enabled remote sensing that is low-cost, highly mobile, and is being treated as an emerging tool that expands the SHM technology inventory [22,23,24]. This further corroborates the necessity of combining WSNs and UAV-based remote sensing technologies.

Reflecting on the trends in PA, SHM, and other similar geospatial-scale monitoring applications for critical missions, we have proposed and developed a prototype to realize a wireless aerial imaging and ground-sensing network; in short, it is termed the aerial–ground wireless sensing network (AG-WSN) [25]. First, this AG-WSN features the use of one or multiple UAVs as the primary imaging nodes, which will serve as the gateway to the ground sensors; second, the wireless sensing units are deployed (by UAV delivering or manual installation) in the ground (or ground structures) over a geospatially large or a spatially complex space. The combination of low altitude imaging and ground sensing provides the power of fusing remotely captured images with a high resolution and point-based ground-truth data in the field. The high mobility of the UAV allows it to be deployed opportunistically according to scheduled tasks or in response to unexpected urgencies (e.g., disasters). Combining the collaborative aerial and ground sensing and the opportunistic operation modes (e.g., a ground node may be only active when the UAV hovers above it and collects data from it), we state that the proposed AG-WSN can potentially provide the most high-fidelity and most flexible sensing solutions to many monitoring problems arising from the need to assess geospatially large and complex built/agriculture environments.

In this paper we first address the opportunistic nature of the AG-WSN by reviewing the related UAV-WSN integration efforts and proposing a conceptual operation design, which further motivates the proposition of sensor activation for network energy efficiency. Centering around sensor activation, we propose to develop a sensor wake-up solution. Related work is reviewed that shows the benefits and drawbacks of different wake-up designs and the rationale for choosing an active radio frequency (RF) mechanism. Subsequently, a general out-band RF wake-up mechanism is developed and demonstrated. For a comparative purpose, the infrared wake-up prototype is implemented too. We further conduct a comprehensive study on energy conservation, followed by the conclusions and remarks for this paper.

The scholarly contribution in the design of this work is two-fold: first, it provides a realistic design of active RF-based wake-up mechanisms for sensor activation in a novel aerial–ground sensing network (AG-WSN), which bears the goals of rapid prototyping and having a realistic application in urban- or farming-scale monitoring tasks; second, the experimental evaluation reveals the superb effectiveness of the proposed wake-up method and its appropriateness for the AG-WSN system. The technical contribution in the experimentation of this work also includes (1) the use of a digital imaging approach to measure the wake-up time delay and (2) the resulting time-dependent rates of the battery-based power consumption using three different wake-up methods.

## 2. Unmanned Aerial Vehicle Wireless Sensing Network (UAV-WSN) Integration, Opportunistic Sensing, and Research Needs

To our best knowledge, there were only a few efforts that attempted to integrate UAVs with wireless sensor networks. In [26], UAVs were considered as mobile sinks for ground sensor data dissemination. This approach intended to optimize the route from a given sensor node on the ground to a few mobile sinks that move in the area. Authors in [27] presented a different approach that kept the sensor network continually connected. It used multiple UAVs to establish a reliable relay network to guarantee the delivery of data produced by the wireless network nodes on the ground to the users. Given these few simulation-based and conceptual efforts, even fewer efforts are found that physically realized a UAV-based sensing network system. In a recent effort, the authors developed a WSN using a fixed-wing UAV as the aerial gateway for marine data collection [28]. In our recent effort, we further investigated the interference between the WiFi-based video transmission link and the ZigBee-based ground-data transmission links [25].

The use of flying single or multiple UAVs, either as a mobile sensor node or a data sink, triggers the need to optimize network efficiency between sensors and sinks. Energy cost is an inevitable constraint, considering that both the UAVs and ground sensors are, to date, usually battery-powered. An opportunistic network is the emerging technology that solves such an optimization problem. In [29], protocols were proposed to better exploit the durations of high-quality channel conditions. Based on that, authors [30] proposed routing protocols that increase the throughput of large unicast transfers in a multi-hop wireless network. There are also a number of research efforts on optimizing resource and performance in wireless sensor networks. In [31], the authors considered a different scenario where the paths from message sources to their destinations do not always exist. Then the authors analyzed protocols that alleviated the problem of chronically disconnected paths by having a node store the packet, carrying it until meeting another relay node and forwarding the packet to the other relay node. In a more recent effort, researchers also developed middleware that implemented the opportunistic network into mobile social networks [32]. It is noted that for general opportunistic networking systems (without using a UAV as a gateway node), different protocols are proposed, including the flooding protocol and the history-based protocol (e.g., [33,34]). Another possible approach at a higher level is to apply the software defined network (SDN) concept into an opportunistic system. In a recent work [35], the author proposed an energy efficient SDN framework for wireline–wireless cross networks, which can potentially be helpful for large-scale deployment. Last, it is stated that these optimization or software-based schemes mostly focus on designing improved communication protocols by assuming that either the UAVs or the sensors are not constrained by the battery-based power.

**Figure 2:**
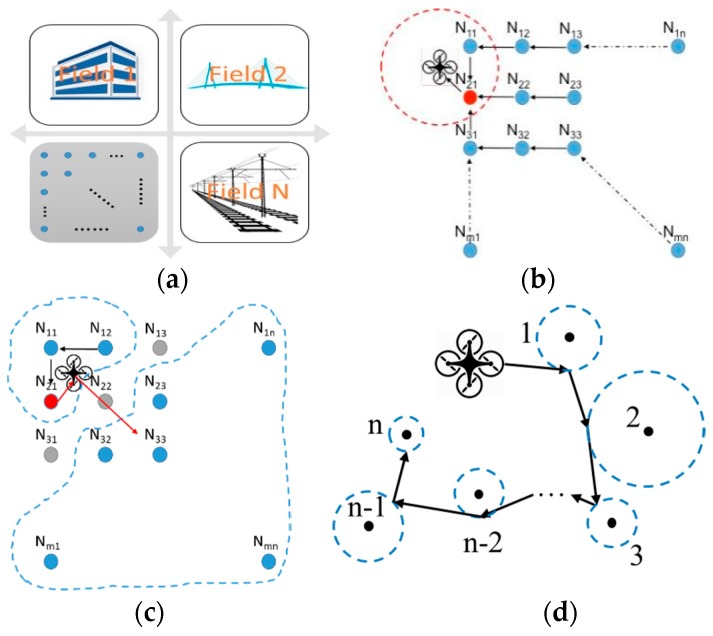
Conceptual field development of an AG-WSN and opportunistic networking: (**a**) sample field sub-networks; (**b**) a sub-network case wherein all sensors work normally and the UAV receives data directly or via relay without flying to individual sensors; (**c**) a sub-network case wherein some sensor failure is allowed and the UAV needs to be mobile to collect data; and (**d**) a sub-network case wherein the sensors are far with each not allowing relay, and the UAV needs to be fully mobile to fly to individual sensors for data collection.

To illustrate such energy constraint, Figure 2 illustrates a conceptual AG-WSN, where (besides being the imaging and computing hub) the UAV is designed as a robotic vehicle that flies to ground sensors at tactical locations. This operational mode, and furthermore, the possible loss of sensors, sensor malfunctions, and out-of-range communication, renders the underlying network as opportunistic, which affects energy consumption in the UAV and sensors.

In Figure 2a four subnets are shown, which indicate four physically isolated sensor networks in the fields, except that the UAV can fly to each subnet to execute opportunistic sensing. Figure 2b indicates the idealized situation where sensor failure (or other malfunctions) and energy consumption does not need to be considered. Hence, assuming each node N_mn_ can communicate with its neighboring nodes N_m±1,n±1_ through a wireless connection, when the UAV flies into this sub-network some of the nodes are in the communication range of the UAV (within the red dashed-line circle), while some are not. The UAV will pick one of the nodes in the range as a relay node, herein which is N_21_, and collect data from any other node in this subnet. Figure 2c illustrates when sensor failure happens; the UAV can move from one disconnected zone to another one. Figure 2d demonstrates a different scenario where all sensor nodes are far away from each other and no wireless connection can be established between them. In this case, the UAV will reach within each node’s communication range to communicate with individual nodes. Then, the existing optimal communication protocols can be used.

When the energy consumption of either the UAV or sensor networks is considered, optimization in the physical layer (rather than in the communication protocols) needs to be addressed. Two apparent scenarios exist: (1) through spatial path-energy optimization the UAV finds the optimal flying path through the geospatially deployed ground sensors, for which it belongs to a typical traveling salesmen problem (TSP) and is being tackled in our recent research [36]; and (2) through a sensor activation approach, as being concentrated on in this paper, such that sensors are only active when the UAV is in its neighborhood.

## 3. Sensor Activation and Related Work

Although solar power or other intermittent energy supply techniques exist, battery power continues to be considered as the most reliable source for powering sensors and robots. By implementing the commonly adopted duty-cycle method, wireless sensor nodes could be pre-programmed to wake up and communicate with the gateway, then go back to sleep after communicating. This approach for extending battery life has been treated as a default function in many commercial wireless sensors. A number of researchers also attempted to optimize power management to further extend the battery life of WSNs [37,38,39,40]. However, one key problem that prevents us from realizing long-term aerial–ground sensing is the opportunistic nature of deploying the UAV (gateway) and the sensor nodes. In an AG-WSN, the gateway (as a payload of the UAV) is deployed spatially to proceed toward the ground sensors on a non-scheduled basis or randomly upon abrupt events. This further implies that the ground sensors do not have ‘knowledge’ or are not programmable to realize duty-cycle sensing. If the ground sensors are turned on to include at least the microcontroller and communication units (whereas the sensing units may be on or off according to the duty cycles), the battery of the sensor nodes may be drained quickly.

One straightforward approach to such energy inefficiency issues is to wake up ground sensor nodes when the UAV is deployed as needed to approach the sensors without any preprogramming. In this paper, we first propose to use a radio frequency (RF)-based out-of-band wake-up mechanism. Then, comparative studies are conducted to investigate their energy saving performance against two other wake-up mechanisms. Using a traditional star-like sensor network, the analytical and experimental studies show solid evidence that the RF-based wake-up mechanism outperforms the other two solutions on energy consumption.

Earlier efforts reveal that data transmission in a WSN is generally very expensive in terms of energy consumption, whereas data collection (or the sensing itself) consumes significantly less [41]. For this reason, various methods are developed to extend the life of battery-powered WSNs by reducing the power consumption of the wireless modules. A significant number of efforts were found that focused on developing lower level network protocols by adopting duty-cycle based solutions [42,43,44]. These studies aimed to optimize the network protocols, specifically through reducing the energy consumption during the idle or the listening time of the wireless modules. For example, the authors in [45] proposed an adaptive medium access control (MAC) protocol, which introduced a flexible duty-cycle method and claimed to reduce 96% of energy use compared with traditional protocols. However, the core concern for these duty-cycle solutions is that the wireless modules do not know when the data transmission is coming or required, and the node must listen periodically to limit data latency; thus, the duty-cycle ratio cannot go arbitrarily low [46]. Also, duty-cycle methods may have problems with delay and synchronism and, hence, the protocol is relatively complicated. As such, using waking-up mechanisms to answer this concern has been extensively studied.

A number of sensor activation methods have been proposed to date. Essentially, such an activation approach features a waking-up mechanism for activating sensing modules in an as-needed (or on-demand) basis. There are two categories of methods when considering wake-up mechanisms for use in wireless networks: in-band and out-band. If an in-band method is used, a special value is transmitted through the data channel to send out the wake-up signal. By contrast, a separate channel is needed to realize such a waking-up mechanism in an out-band method. Using in-band methods can reduce the complexity and cost of the implementation. A recent study on the in-band wake-up method [47] claimed that, by using both game theory and reinforcement learning techniques, it achieved very effective sleep/wake-up scheduling. However, it kept the wireless communication channel busy and may have required more energy consumption. From an energy-efficiency perspective, the out-band approach is more suitable for the proposed concept that emphasizes opportunistic aerial-ground sensing.

There are many studies that employ the out-band wake-up mechanism [48]. In this paper, they are categorized into two groups according to their communication medium: (1) non-RF based and (2) RF-based. In a non-RF based mechanism, researchers proposed wake-up methods using infrared (IR), optical, and acoustic signals. The authors in [49] developed an IR LED-based wake-up mechanism, in which the receiver was a photo-detector that received IR signal and then generated an interrupt. The authors stated that their IR design only consumed 12 μW while listening. It is noted that the obvious drawback of this prototype is the sensitivity of the circuits to external light and vulnerability to ambient noise. In [50], the authors presented a home-energy management system using infrared, signal-based control over a Zigbee network. In this system, an infrared receiver was attached to the Zigbee gateway. The Zigbee gateway was responsible for communicating with other home appliances, whereas the infrared remote control was the out-band wake-up channel used to wake up the Zigbee network. Unfortunately, this paper did not mention the power consumption of the IR receiver. To our understanding, this type of IR receiver in the paper is commercially available and similar to the one used in our experiment, as shown in this paper, which has a better resistance to noise at the cost of a much higher power consumption, and it may require up to 45 mW according to our experiment.

Optical communication is another non-RF option for the secondary wake-up channel. Two efforts in [51,52] used free-space optical (FSO) communication as the transceiver. The receiver, at idle listening, consumes 317 μW and 695 pW. However, the transceiver and receiver both need to be placed in line-of-sight (LOS), and the data rate is slow. It is impractical for use in a UAV since it mostly does not stay in a position that accurately faces the transmitter. Thus, this option is not suitable for our application. The AG-WSN scenario may also limit the use of acoustics as wake-up methods [53,54] due to the noise produced by the UAV blades. Ultrasonic, as stated in [55,56], may avoid the noise made by the UAV. It uses a piezoelectric transducer that converts the mechanical energy into electrical energy for generating wake-up interrupts. However, most ultrasonic communication and ranging efforts, to date, are applied in indoor (short-range) or LOS scenarios [57,58].

In comparison to the non-RF based wake-up mechanisms reviewed above, RF-based communication has the advantages of not requiring LOS, it has better noise and interference tolerance, a higher data rate, and it is more cost-effective. Research on the RF-based wake-up mechanism can be divided into two designs: passive wake-up and active wake-up, both of which have been well studied in the laboratory environment. In a passive design, the RF receiver harvests energy from the transmitter to power itself, thus, requiring no power supply [59,60,61]. In [62], the authors simulated a passive RF wake-up receiver, in which they indicated that comparing with the existing duty-cycle method, their RF wake-up could significantly enhance energy efficiency by up to 70%. There are also simulations on both passive and active RF wake-up circuits, such as [63,64]; the authors of these efforts later implemented the passive RF circuit into a sensor network with a multi-hop capability [65]. However, among these passive RF-based methods, information on the communication range between the transceivers was not found. In addition, it was reported that the harvested energy by the receiver may decrease with increasing distance between the receiver and the transmitter. The authors in [66,67] showed that the hardware setup could only reach a maximum distance of 4 m for a successful wake-up. Considering the AG-WSN scenario proposed in this work, the passive RF-based wake-up design is not suitable.

Regarding the active RF wake-up design, as mentioned in [46], there are 13 active RF-based wake-up methods using discrete components, whereas there are 29 methods using complementary metal–oxide–semiconductor (CMOS technology. The most significant parameters relevant to these designs and prototypes for the interest of the proposed AG-WSN configuration are power consumption, range, address decoding capability, wake-up latency, and balancing. For example, the author in [68] configured the wake-up receiver using discrete components and claimed to achieve 120 m of communication range. However, the receiver consumed 1620 μW at the state of idle-listening, which is too high for the battery-powered nodes. There is a low-power design in [69], which only consumed 52 μW. Unfortunately, the authors did not provide a range test. A favorable design was presented in [70] recently. It achieved a communication range of 50 m at idle-listening with a power consumption of 1.2 μW. Unfortunately, at the time of our experiment, there was no market-ready product or porotype based on this design.

In this paper, the use of off-the-shelf components is stressed in our prototyping and experimental validations, with the goal of rapidly putting the proposed AG-WSN into practice. As many researchers have similarly done, the AS393X wake-up receiver has been used in many efforts and designs [71,72,73,74,75]. Among these researchers, the author in [74] used AS3933, which is the same chip in our experiment, to prototype the receiver circuit to have an 87 m communication range, at the cost of more than 5000 μW of power consumed when decoding the wakeup signal. Also, in a recent paper, the authors compared the RF wake-up mechanism and the low-power listening techniques [76]. They concluded similarly what we achieved in our energy evaluation results in this paper. However, the authors of this paper did not measure the delay caused by the RF wake-up transmission, and their power consumption measurement was not based on batteries, but a constant power supply, hence, it lacked a realistic configuration.

## 4. Proposed Energy Efficient Sensing Network

### 4.1. Topology and Implementation

In our aerial–ground approach, the UAV was the wireless network gateway, which was responsible for communicating with each individual sensor node that was deployed in the field. Although there are many wireless protocols that can be configured for these sensors, we chose the XBee (a modified Zigbee wireless protocol) wireless module for constructing the network, since its power consumption is relatively low. The XBee protocol allows three types of network topologies, which include: star, mesh, and cluster-tree. Although mesh and cluster-tree networks have a very flexible network structure, they both require some sensor nodes in the network for relaying data to the gateway. This means that these relay nodes have to either always be active or be duty-cycle active, further consuming a significant amount of power. Alternatively, the star topology does not require any node to relay information, and they can be kept in sleep mode for most of the time. When using the star topology, the UAV will be the XBee coordinator (gateway) for collecting data from multiple ground nodes in the communication range. More importantly, the high-mobility UAV will wake up multiple ground sensor nodes from their sleeping state based on the demand of the UAV, which can fly overhead to perform sensor activation and data collection on an individual or group of sensor nodes. Therefore, the star topology was considered the most appropriate topology for our proposed aerial–ground network.

One concern is that using UAV as a flying gateway may consume more energy per UAV flight than what could potentially be saved in our wake-up mechanism. In most cases, the batteries in sensor nodes are hard to replace due to a variety of reasons, e.g., the position of the node may be difficult to reach, or the battery is sealed in a box and buried in soil or within structures to avoid harsh weather conditions. The key concept of our approach is to reduce the consumption of the sensor-node batteries in the field as much as possible to sustain service times as long as possible. For the power consumption, we state that in reality multiple identical UAVs with multiple high-capacity battery backups can be used to perform their functions in remote sensing and serving as the network gateway.

Last, for implementing the sensing nodes, we use Libelium’s Waspmote^®^ (Zaragoza, Spain) as our ground sensing nodes [77]. The Waspmote sensing node contains a low-power MCU, embedded sensors, and optional wireless module slots. It consumes quite a small amount of energy when its sleeping mode is selected.

### 4.2. General Active Out-Band Wake-Up Mechanism

**Figure 3:**
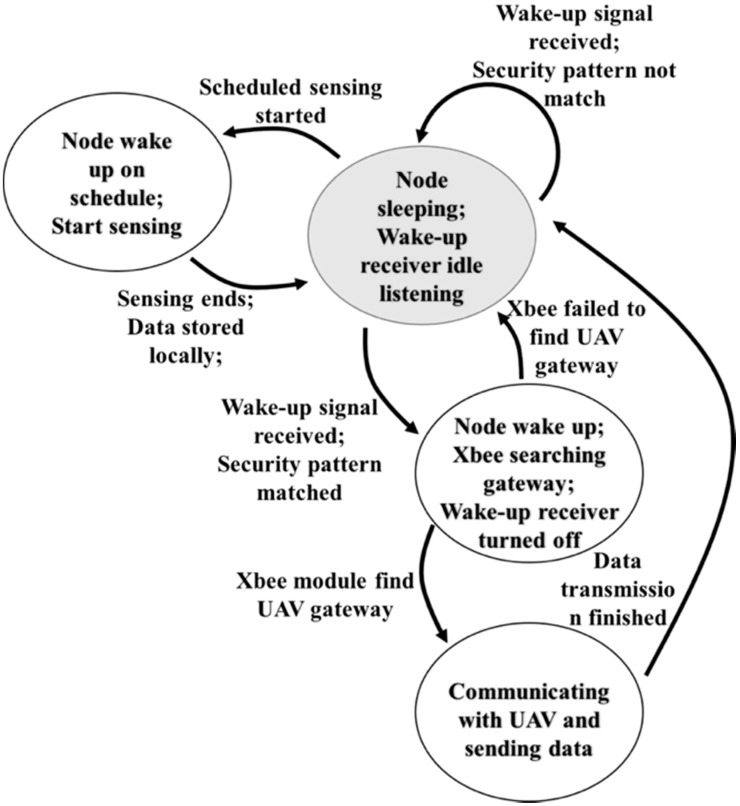
State and action diagram of the ground sensor nodes and the unmanned aerial vehicle (UAV).

As reviewed earlier, an active, out-band wake-up mechanism was chosen for the proposed AG-WSN. To understand this mechanism, Figure 3 summarizes the state and action diagram for the wake-up mechanism and the sensor-node operation. A description is given as follows:

(1) When the sensor node is deployed in the field it is pre-programmed with a duty-cycle sensing schedule. It starts off in sleep mode, which we call the initial state. Only the wake-up receiver is listening in our example, which is either an infrared or RF wake-up receiver.
(2) If a wake-up signal is received by the sensor node, it will check whether the signal matches the pre-stored pattern. If not, the node ignores the signal and changes back to the initial state.
(3) If the wake-up signal matches the stored pattern the node will wake up, start the XBee module, and turn off the wake-up receiver. Then, the XBee begins scanning the gateway on the UAV.
(4) If the XBee module fails to find the gateway in a couple of tries, the node shuts down the XBee module and returns to the initial state.
(5) If the XBee module successfully connects to the gateway, it starts communication with the UAV as programmed (e.g., sensing data or updated schedules).
(6) After the communication ends, the node returns to the initial state again.

**Figure 4:**
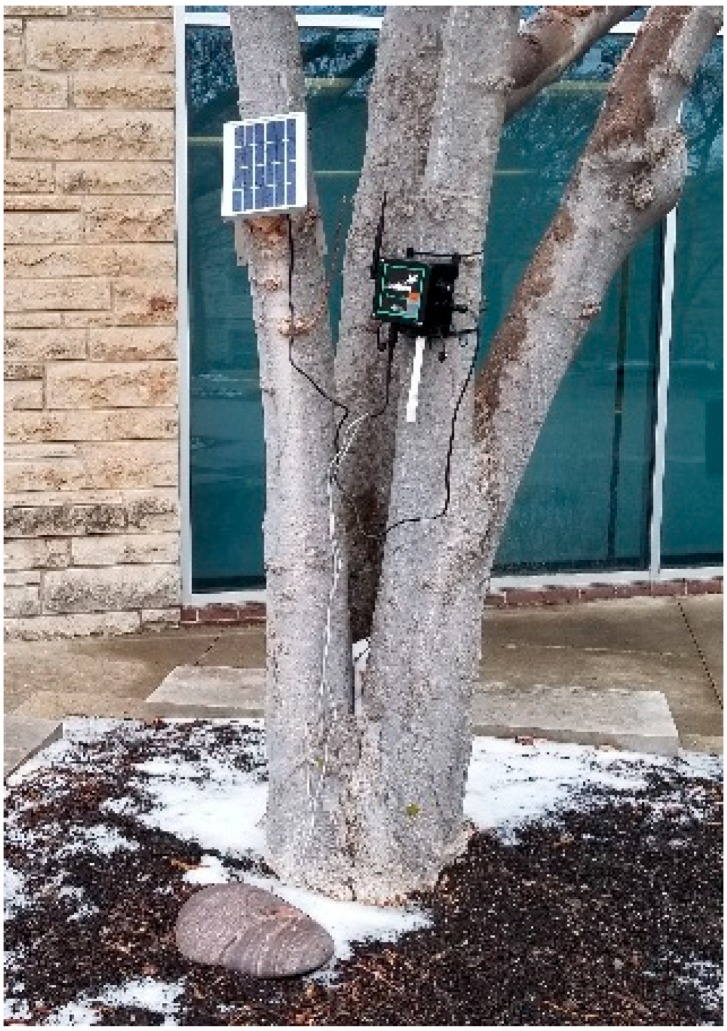
An actual WSN deployed in the field, using the same sensor modules tested in this paper.

It is noted that in this design the sensing cycle is preprogrammed and may not match the wake-up cycle when the UAV is approaching. This is because the sensing cycle happens more often than the UAV data collection cycle. In a practical application such as in an SHM or PA sensing system, these sensors may be pre-programmed to wake up serval times a day to sense the ground-truth data, while the UAV only approaches these individual sensor nodes once a day, or even less frequently in the case of flying on demand. In this case, the sensor nodes can store the data locally and send all the data to the gateway once the UAV wakes up the nodes. Although this would possibly force the sensor nodes to send massive amounts of data in practice, we find the data sizes vary significantly in different applications. One example is the environmental monitoring project, as shown in Figure 4. In this garden monitoring project, the sensor nodes included the modalities of soil temperature and moisture, atmosphere pressure, humidity, and temperature. The results showed that each sensing cycle produced less than 500 bytes of data to transmit with the sensing cycle setup at every hour, and the total data collected from one sensor node was less than 12 K bytes. In another test the vibration sensors were attached, which required the sensor to run for 1 min at 20 Hz for each cycle. The data produced in each cycle was about 5 K bytes, which led to a total of 120 K bytes of data for a single day if the sensing cycle was set to every hour. That said, the Xbee module adopted in this work claims to have a maximum data rate of 30 K bytes/s, which is fast enough for the UAV to collect all the data from the sensor nodes.

## 5. Radio Frequency (RF) and Infrared Wake-Up Mechanisms and Implementation

### 5.1. Proposed RF Design and Implementation

**Figure 5:**
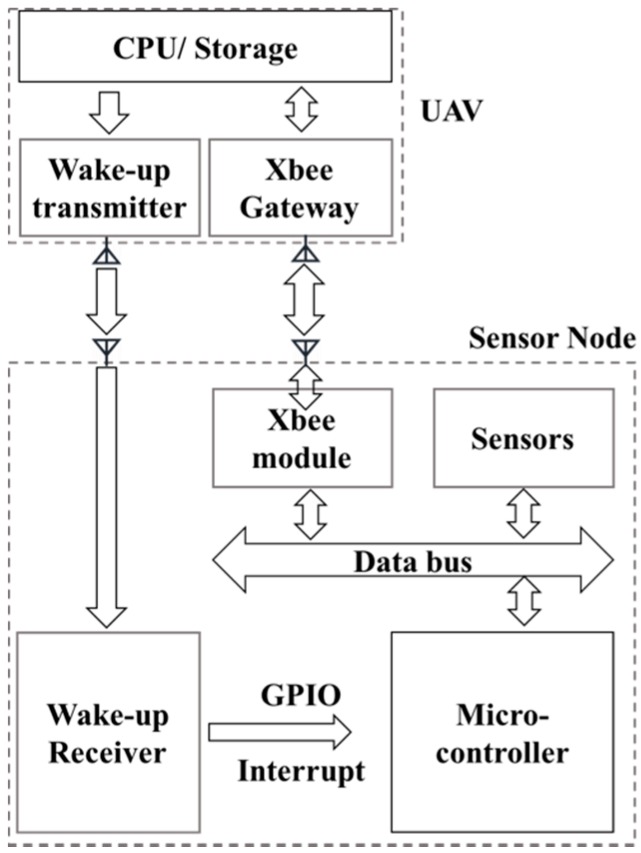
Design for the radio frequency (RF)-based active wake-up mechanism.

The RF wake-up approach is the latest innovation towards achieving energy saving for wireless networks. There are two types of implantation methods: (1) the method that uses active wake-up receivers and utilizes energy from a battery, and (2) the method that uses passive wake-up receivers and harvests energy from the wake-up radio. In our approach, we find that active wake-up receivers have a much better performance in range and a higher rate of success in changing between listening and wake-up modes. In addition, the energy consumption for the RF receiver can be as low as serval µW while it is in its listening state. Figure 5 summarizes the RF-based wake-up design proposed in this paper.

**Figure 6:**
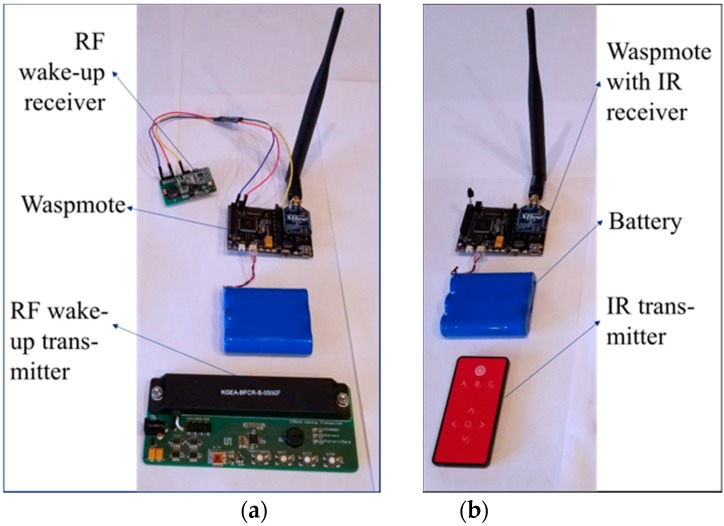
Hardware components of the two wake-up systems: (**a**) RF wake-up and (**b**) infrared wake-up.

To implement the design in Figure 5 we choose to use a commercial product, AS3933, as the wake-up receiver, which is further attached to the Waspmote sensor node. AS3933 is a 3-channel low power amplitude-shift-keyed (ASK) receiver, which is able to generate a wake-up signal upon detection of a data signal that uses an LF carrier with a frequency range of 15–150 kHz. The receiver’s output is connected to the MCU’s interrupt pin at the sensor board. When the AS3933 receives an RF signal, it decodes and checks whether the signal matches the pre-stored pattern. Once confirmed, the receiver will send out a pulse to the interrupt pin to wake up the MCU. Figure 6a shows the hardware setup of the three components in our RF wake-up prototype.

By adding the RF antenna to the UAV and programming the micro-controller on the UAV to generate a Manchester wake-up pattern, we were able to use the UAV as a control hub to wake up sensor nodes in range. Since the MCU was in sleep mode using this out-band wake-up method, one interesting question was how long it would take for the MCU to wake up after the wake-up signal was sent out. If this procedure took a significant amount of time, then we needed to consider this delay as a drawback for this RF wake-up mechanism. This potential pitfall is carefully studied in this paper.

### 5.2. Infrared Wake-Up Implementation

To achieve a comparative setup to justify the proposed RF method, the infrared wake-up mechanism was also implemented in this endeavor. Infrared is a commonly used solution for simple wireless communication at short ranges [48]. There are two basic components in infrared wireless communication: emitter and receiver. The emitter first transmits the coded data, generated by the micro-controller, to the receiver. When the receiver reads the IR signal, it decodes the signal into digital data and then passes the information to its following component, i.e., the sensor node. In our implementation we integrated a 950 nm IR LED into our UAV and a TSOP38238 IR Receiver Module on the sensor board. Figure 6b shows these modules. The emitter was connected to the UAV with the MCU’s PWM-capable I/O pin, and the receiver was connected to the MCU’s regular digital I/O pin on the sensor board.

To achieve the wake-up function, the emitter on the UAV sent out the wake-up signal to the receiver only when the signal matched the code that was stored in the MCU (on the sensor board). The sensor board then turned on its sensors and the XBee communication module. In this setup, the sensors and the XBee module could be turned off until the MCU received the wake-up signal. However, the sensor board MCU always had to stay on in order to check whether the infrared signal matched the specific pattern.

## 6. Experimentation and Results

In the following we evaluate the wake-up range as well as the energy consumption for both out-band methods (RF and IR), then compare the energy costs with the traditional duty-cycle method, all based on the same hardware setup.

### 6.1. Physical Verification and Comparison

Infrared communication was directional since the wavelength was close to visible light. We used a 950 nm infrared LED as the transmitter, and TSOP38238 as the IR receiver diode. In the test, we placed the IR receiver on the ground, facing up. The UAV carried the infrared LED hovering above the receiver and continued sending signals. It was found that the effective maximum range of the IR communication was highly affected by the environmental disturbances and the variations in the supply voltage. Under strong sunlight, the IR receiver could still read signals from the LED; however, it often failed to decode the pattern in the code when the communication range exceeded 3 m by applying a 3.3 V power supply to the receiver. We found that by increasing the supply power of the IR receiver to 5 V, this issue was resolved. After multiple tests, we concluded that with our hardware setup, 5 m was a safe distance for the IR communication in an LOS space without obstruction and with a 5 V power supply for the receiver.

### 6.2. RF Wake-Up Distance

**Figure 7:**
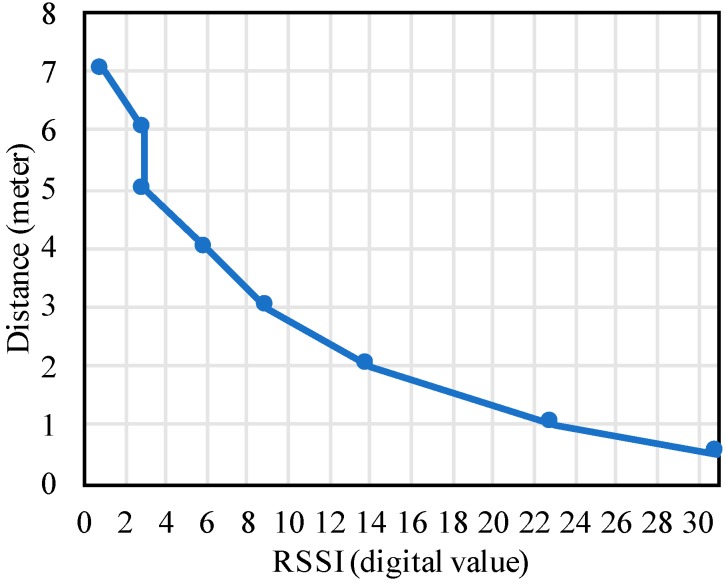
Received signal strength indicator (RSSI) of the receiver at different distances from the transmitter.

RF wake-up, however, did not require a direct LOS. The receiver, AS3933, had three wake-up channels, each connecting to an antenna. In our test, the three antennas were set up in a perpendicular fashion. Any antenna that received the correct pattern may trigger the wake-up. To test the wake-up range, we used a 125 KHz antenna connected to the UAV’s MCU and external power supply. When the supply voltage for the transmitter was set to 9 V, the maximum range was around 7 m in an indoor environment. A similar result in the outdoor test using the same receiver was found in [71], which resulted in a 5 m range using a 12 V supply for the transmitter. One question related to the wake-up distance was whether the power consumption would increase as the distance became greater. To get more detailed data on the wake-up distance and its energy consumption, a ground test was implemented where the position of the same transmitter was fixed, and the AS3933 receiver was moved away from it. We recorded the received signal strength indicator (RSSI) of the receiver at different distances, and the data is shown in Figure 7. The RSSI was measured in digital values with a 5-bit accuracy. The relation to the input voltage provided by the AS3933 manufacturer shows an approximately a linear relation [78]. Combining our testing result in Figure 7 and this information, they gave us a general idea that how far the receiver could work and what the voltage was at the AS3933 input.

**Figure 8:**
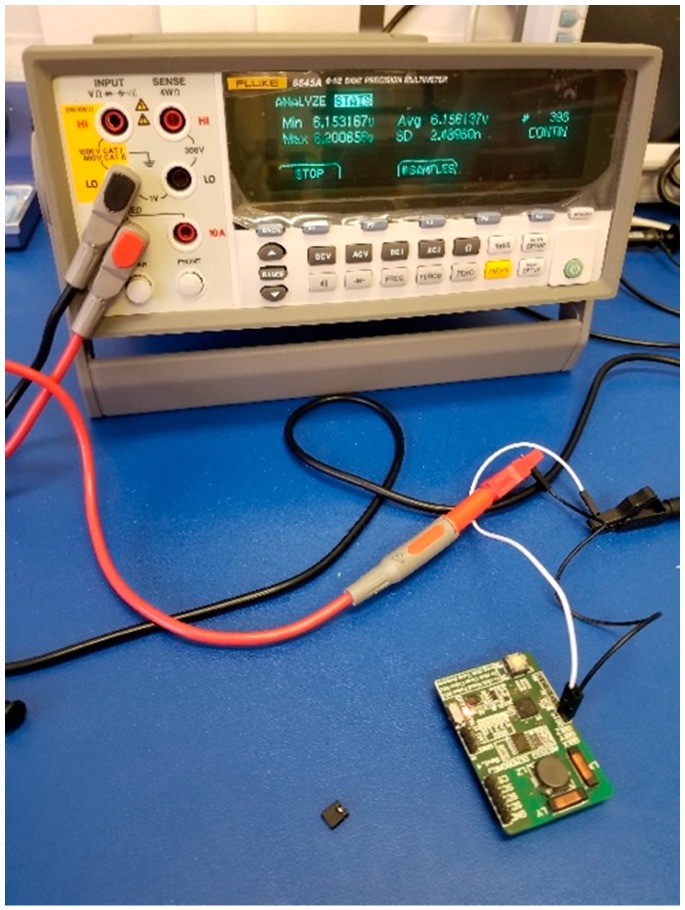
Current measurement of the AS3933 in a standard listening mode with three active channels and the RC-oscillator as the clock generator.

Following the RSSI test, we used a 6.5 precision meter, Fluke 8845A, to measure the power consumption under these distances. In this setup, we programmed the receiver as a standard listening mode with three active channels and used the resistor–capacitor (RC) oscillator as the clock generator. From the receiver’s manual [79], it showed the typical current was 6.1 μA, which was also confirmed by our measurement (Figure 8). Then, we measured the current of the receiver while it was decoding the signal from the transmitter. The results are shown in Table 1. It was found that as the distance increased, the current of the receiver increased. This was mainly because as the distance became further, the RSSI value lowered as discussed, and the receiver’s variable gain amplifier increased the gain; therefore, the signal could be decoded properly. We also observed that, interestingly, within a 2.5 m range the receiver’s working duration remained around 200 ms. Once the range passed 3 m, the working duration increased to 460 ms, which indicated that more power was consumed.

As the voltage for the receiver was 3 V, the results above indicated that the lowest possible energy consumption was 4.632 μJ (at 0.5 m), while the highest was 13.053 μJ. Considering that most of the time the receiver was in a listening mode with the maximum current of 6.1 μA (with 3 channels on), and further assuming that the listening time was 30 min before a wake-up signal arrived, the energy consumed during the listening states was 32.94 mJ. Compared between 32.94 mJ and 13.053 μJ, the increased energy due to a larger distance can be ignored.

### 6.3. RF Wake-Up Delay

Since it took time for the MCU to wake up from sleeping, we expected that this may cause a delay in the data gathering for the WSN. Other wake-up receiver designs, like the one reported in [80], claimed to have a latency of 214 ms, while in [70], the author achieved around 0.9 ms. We wanted to compare our setup with other wake-up designs to make sure the latency value was in an acceptable range. To evaluate this and measure the time it took for the signal to reach the MCU after sending a signal from the transmitter, we set up a novel photogrammetric test environment. In the RF wake-up setup we had three different components: the wake-up transmitter, the wake-up receiver, and the MCU. They are introduced in Figure 6a The measurement operates as follows:

(1) When the transmitter sends out the wake-up signal, the LED on the transmitter will flash. This moment is defined as t_1_.
(2) When the receiver receives the signal and decodes it if the signal matches the pre-stored key, then the LED on the receiver will flash. This moment is t_2_.
(3) When the receiver sends out the wake-up trigger to the MCU interrupt pin, then the MCU wakes up and the LED on MCU board will flash. This moment is t_3_.

**Figure 9:**
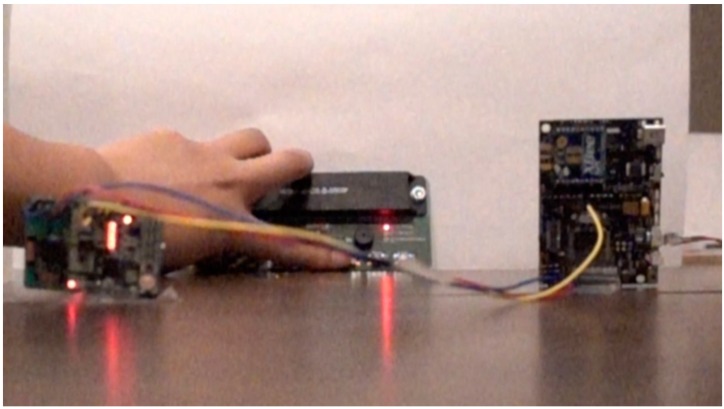
One image captured in the high-speed video. In the center is the wake-up transmitter, on the left is the wake-up receiver, and to the right is the microcontroller unit (MCU). This image is the frame when the receiver decodes the signal and finds that it matches, thus, the LED on the receiver board is lit up.

We used a high-speed camera (Sony RX100 V), which can record 1000 frames per second, to record the above sequence in a video format. We can obtain the time delay by calculating the video frames between each LED light up, as each frame is equal to 1 ms. One picture frame of the video is shown in Figure 9.

After the test, we found that the receiver had some failed wake-up instances due to environmental RF noise when simple coding (short pattern) was used. The solution to this would be to increase the wake-up single pattern from 16-bit Manchester coding to 32-bit, or double the wake-up single pattern length. Four coding conditions were considered and tested, and the time intervals between t_1_ and t_2_ and between t_2_ and t_3_ were recorded. Combining all these coding solutions, the time delay values averaged over multiple tests are reported in Table 2.

The above results reveal that with different coding setups for the transmitter and receiver, first, the time delay between t_1_ and t_2_ increased when longer-bit Manchester codes or double patterns were transmitted. This was because both transmitting and decoding phases took longer if the coding was more complex. Second, the delay times between t_2_ and t_3_, as shown in Table 2, stayed comparatively the same. This was because t_2_ was the time when the wake-up receiver sent out the signal through wiring to the MCU, when all decoding procedures were already completed. Thus, the delay between t_2_ and t_3_ only represented the wake-up time of the MCU and would not be affected when different coding patterns were used.

Regardless, since the total delay caused by using this RF wake-up mechanism was less than 80 milliseconds (from sending out the wake-up signal by the transmitter to the MCU’s wake-up), we safely concluded that this wireless wake-up mechanism did not affect the data communication between the gateway and the sensor nodes in the field for most sensing applications, specifically when the sensors sense data packets at one time and transmit at a different time, then stay idle with much longer durations. The study in [81] claimed to reduce the time between t_1_ and t_2_ roughly from 13 ms to about 2 ms using 16-bit and a single pattern with a similar setup in this paper. We thought this could potentially further reduce the latency introduced by this out-band wake-up mechanism. Another study in [72] using the same chipset claimed the time between t_2_ and t_3_ was 45.87 ms, which was similar to our experiment.

### 6.4. Energy Consumption Analysis and Verification

Energy consumption was one of the primary concerns of this paper. Our main study focused on energy consumption in the sensor nodes that were potentially deployed in the hard-accessible field. Specifically, we classified each sensor module hardware into four subunits represented by the primary device: the MCU, XBee module, wake-up receiver, and the sensors. In Table 3, the typical current consumption with a 3.3 V supply voltage for this hardware is listed. We did the comparative experiment on one default (duty-cycle) energy-saving mode and two wake-up mechanisms, as explained earlier, and recorded the power consumption:

(1) Solution 1―the simple duty-cycle method (default in the Libelium sensor network). In this method, no out-band wake-up method was used. We used the XBee as our data communication as well as the wake-up channel. The XBee module on the sensor node stayed in listening mode when no UAV was nearby. Using this solution, the XBee and MCU on the sensor board had to always be turned on.
(2) Solution 2―infrared wake-up method implemented in this paper. The infrared receiver was used as the wake-up channel. Since the IR receiver was connected to the MCU GPIO, it required the MCU to always stay on, while the XBee module could be turned off.
(3) Solution 3―RF wake-up method proposed and implemented in this paper. The RF was used as the wake-up channel. When the receiver was connected to the MCU’s interrupt pin the MCU stayed in sleep mode. The MCU only required 55uA of current while in sleep mode.

**Figure 10:**
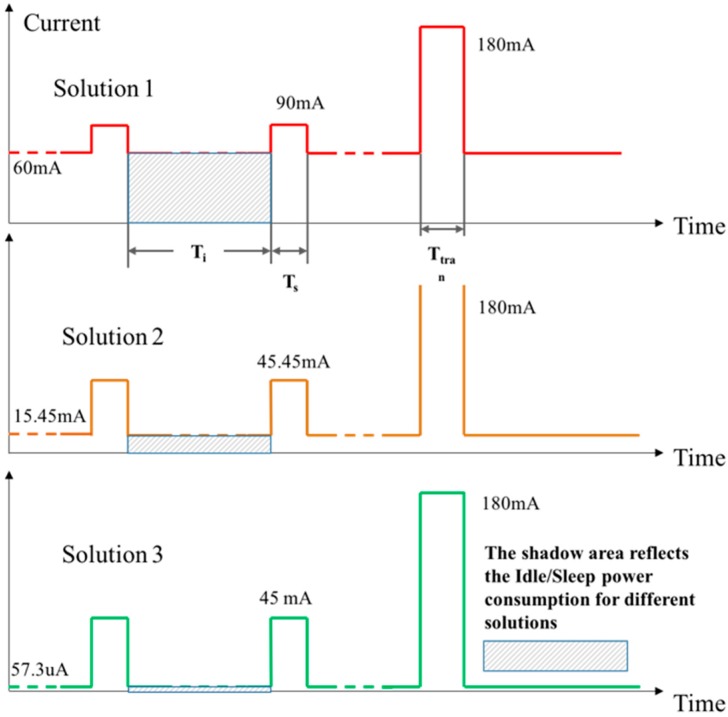
Energy consumption illustrations resulting from the three solutions.

We assumed the same schedule for the different solutions, in which T_i_, T_s_, and T_tran_ represented the duration of the idling state, sensing state, and the transmitting state of the sensor node, respectively. Considering 24 h as a working period, if every 4 h the node sensed the data and transmitted to the gateway, the values of T_s_ and T_tran_ in 24 h were usually less than 10 min, and the remaining time belonged to T_i_. This means that the significant part of the power consumption was spent within the T_i_ period when accumulated over time. Figure 10 illustrates the electric current consumption, considering the aforementioned typical durations.

The power consumption for one sensing cycle can be calculated by the following formula:

(1) $$E_{cycle}=[T_{i}\times I_{i}+T_{s}\times I_{s}+T_{tran}\times I_{tran}]\;V_{dc}$$

where I_i, s, tran_ is the current variable at the state of idling, sensing, and data transmitting as defined in Table 3, respectively. The resulting *E*_cycle_ defines the energy consumption in a designated cycle proportional to the constant battery DC voltage. Using this formula and the data from Table 3, we can qualitatively state that Solution 3 needed much less energy for the idle state. By algebraic calculation based on Table 3 and the assumed typical idling (4 h), sensing duration (1 min), and transmitting duration (1 min), Solution 3 required only 1.6% of the energy compared to Solution 1, and 6% of energy compared with Solution 2. This statement has been similarly stated in [11,13]; however, no physical implantation and comparative validation are found in their efforts.

To evaluate the qualitative statement above, we built a sensing network using Waspmote 1, 2, and 3. Each were set up with XBee modules, and the wake-up hardware corresponded to Solutions 1, 2, and 3. For each implemented solution, the mote was attached with a rechargeable 6600 mAh lithium-ion battery. We programmed that when the Waspmote woke up, the MCU will measure the battery voltage level and calculate the current battery percentage.

**Figure 11:**
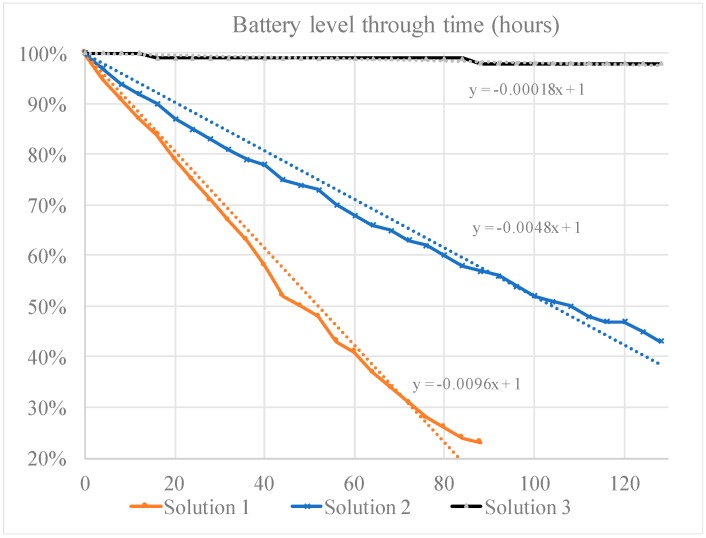
Experimental battery test and capacity dropping for the three different solutions.

A separate XBee coordinator was placed within the line-of-sight to each network of the Waspmote modules, forming a star XBee network in an indoor environment. To compare the difference in energy consumption between these three solutions, we minimized the possible power consumption from the front-end hardware; therefore, no additional sensing units were used in this test. Each Waspmote was woken up by using its corresponding methods and joined the XBee network every 4 h. Then, they sent a ‘hello’ message to the coordinator, which was always turned on. After that, each Waspmote read the current battery level and then went back to its original state: XBee idle listening, IR receiver listening, or RF receiver listening, respectively, as designed and implemented in Solutions 1, 2, and 3. We monitored the test for about 130 h for the three physical prototypes, and the battery levels were measured and recorded. The resulting energy consumption results are collectively shown in Figure 11.

From the above results we found that Solution 1, in which the XBee module and MCU were always turned on, drained the battery quickly, in less than 90 h. The data transmitted in Solution 1 discontinued after 88 h since the battery level dropped to 23%, and the Waspmote stopped working due to the low voltage. In Solution 2, we can still see a linear drop in the battery level, but with a slower rate compared to Solution 1. As we stated above, most energy in Solution 2 was consumed by the IR receiver and the MCU. We concluded that the XBee idle and listening states consumed a large percentage of energy. Although we did not measure until the mote stopped working for Solution 2, we believe the battery level drop was linear, a rate of 0.48% per hour, and would last a total of about 208 h (through linear fitting with a fixed intercept of 100%). Lastly, the battery level of Solution 3 did not drop significantly due to the RF wake-up solution. Due to the low dropping rate, we only measured a 2% drop during the 130 h test. Assuming a linear rate of consumption (through the linear fitting in Figure 11), we expected that the Solution 3 network would continue working for about 5556 h (or about a 7.5-month period). This was a remarkable improvement compared with both Solutions 1 and 2.

Second, all the consumption rates were compared (as shown in the linear fitting in terms of the slope values). One can see that the power consumption rate from Solution 3 was about 3.8% of Solution 2, and 1.9% of Solution 1. This approximately confirms the previous analytical studies. In fact, we noted a better result (3.8% instead of 6%) from the analytical evaluation based on Figure 10 when comparing Solutions 2 and 3. We believe this was attributed to the actual XBee communication time being less than 1 min since there was not so much data being transferred in this test.

Last, the functions obtained through data fitting, as shown in Figure 11, showed approximately linear relationships in all three cases. They may be used as predictive models to guide the planning of battery use and replacement in practice. It is admitted that caution is needed since this test was based on laboratory testing that assumed idealized data sensing and collection conditions. More field testing with real data collection scenarios is needed if an improved battery management strategy is desired.

## 7. Conclusions and Remarks

In this paper we reviewed the concept of using an aerial-ground wireless sensing network (AG-WSN) in a remote and geospatially large, complex space. We particularly recognized the need of implementing such sensing solutions in precision agriculture and structural health monitoring practices. We then recognized the technical challenges in achieving energy efficiency in the ground sensors. Different wake-up mechanisms are then reviewed and compared. Among those mechanisms, we chose an active radio frequency (RF)-based wake-up method and implemented it physically. The focus was on evaluating their performances to achieve energy efficiency in the battery-powered ground sensors. The following findings were achieved through the experimental evaluation in this work:

1. The experimental results in this paper indicated that the RF-based out-band wake-up mechanism can save a great amount of energy compared with the other two solutions (the infrared wake-up and the default duty-cycle methods). A direct comparison between the RF-based solution and the infrared-based solution indicated that the RF-based wake-up mechanism had a noticeably better performance in the wake-up range, and had a tremendous improvement in power consumption. Specifically, the results showed that the RF-based wake-up mechanism could potentially save more than 98.4% of the energy that the traditional duty-cycle method would otherwise consume, and 96.8% if an infrared-receiver method was used.
2. The energy consumption for different RF wake-up distances was evaluated in this paper. The results indicated that as the distance between the transmitter and receiver increased, the receiver consumed more power (around 8.4 μJ). However, it was argued that this value could be ignored compared to the energy consumed in the listening mode, which was at least 10^3^ times higher.
3. The evaluation of wake-up time delay by using a variety of different wake-up signal codes indicated that the time delay was below 80 ms; hence, the delay will not affect most opportunistic sensing applications (wherein the sensors sense the data at one time and transmit at a later time, then the sensors go back to sleep mode until another abrupt event). However, it was pointed out that a stricter time delay evaluation needs to be conducted if synchronization is critical between the sensors.

Given the three findings, it was concluded that the RF wake-up mechanism was the first candidate for implementing the proposed wireless aerial-ground sensing network for monitoring applications in large-scale geospatial or challenging spaces. The technical contribution also included the use of a digital imaging approach to measure the wake-up time delay, and the resulting time-dependent rates of the battery-based power consumption using three different wake-up methods. This experimental and empirical knowledge may be extrapolated in similar sensing network research where sensor activation needs to be integrated.

Last, we point out a few limitations in this effort that warrant future exploration. The first limitation is the RF wake-up range with the device used in this effort, which was relatively short (0‒7 m) compared with the Xbee communication range (about several hundred meters). One practical solution for this is to increase the power of the wake-up transmitter and to consider advanced antenna design (such as using the multiple input, multiple output, or MIMO technology) to increase the sensitivity of the wake-up signal. This may introduce more power consumption on the UAV side. With this potential long-range RF-based wake-up mechanism, the UAV no longer needs to reach the proximity of the sensor nodes. The UAV path planning then becomes a traveling salesman problem with neighborhoods, which was covered in our recent research [36].

The second limitation is the star topology assumed for the ground-level WSN. It is arguable that a mesh topology with communication relays can be used. Potentially, we believe that this decision depends on application context. In our context the UAV had two roles: the gateway to wake up sensors and collect data, and a remote sensing platform to collect geospatial images of the ground. Given this mobility and its dual role, a star topology is considered more appropriate. Nevertheless, if a mesh or other dynamic topology is considered in conjunction with the UAV-based sensor activation, this will demand a new research endeavor. A different scenario that uses the star topology for collecting data in a large geospatial area was discussed in our recent work [36]. In this case, the whole WSN can be segmented into a few subsets with a star topology, and the UAV will stay hovering in each subset for both wake-up and data collection procedures. Once the task is performed, the UAV can fly to the next subset. This practical treatment is believed to be a feasible solution to use the UAV with the aforementioned dual roles in an aerial–ground network in larger outdoor environments.

## Figures and Tables

**Table 1:** The relation of distance between receiver and transmitter, the current of the receiver, and the working duration of the receiver.

Distance (m)	Average Current (uA)	Working Duration (ms)
0.5	8.0	193
1	8.0	198
2	8.2	198
2.5	8.1	198
3	9.4	459
4	9.4	462
7	9.5	458

**Table 2:** Average time delay (in terms of milliseconds) within the wake-up procedure.

Coding Pattern	Time between t_1_ and t_2_	Time between t_2_ and t_3_	Total Time Delay
16-bit, Single pattern	12	49	61
32-bit, Single pattern	18	49	67
16-bit, Double pattern	18	49	67
32-bit, Double pattern	31	48	79

**Table 3:** Nominal current values for subunits within a sensing module.

Hardware	Current
*MCU*	15 mA
*Sensors*	30 mA
*XBee*	165 mA/45 mA *
*IR*	0.45 mA
*RF*	2.3 uA/6.1 uA **

* The two values represent working/idle listening, ** 2.3 μA when 1 channel is enabled, 6.1 μA when 3 channels are enabled.
